# Factors Influencing Person-Centered Care Among Psychiatric Nurses in Hospitals

**DOI:** 10.3390/healthcare12222269

**Published:** 2024-11-14

**Authors:** Ji Su Lee, Mi Heui Jang, Min Jung Sun

**Affiliations:** 1 Major in Psychiatric Mental Health Nurse Practitioner Program, Graduate School of Nursing, Kyung Hee University, Seoul 02447, Republic of Korea; tns0226@khu.ac.kr; 2 College of Nursing Science, Kyung Hee University, Seoul 02447, Republic of Korea; dnfntk0213@khu.ac.kr

**Keywords:** psychiatric nurses, empathy, teamwork, nursing work environment, person-centered care

## Abstract

Objectives: This study aimed to examine the association between psychiatric nurses’ empathy, teamwork, nursing work environment, and the degree of person-centered care, as well as to identify factors influencing person-centered care (PCC). Methods: An online cross-sectional survey was conducted from 11 January to 19 January 2024, using four validated questionnaires. Results: Participants included 167 psychiatric nurses with more than one year of clinical experience working in South Korea. Person-centered care was positively correlated (*p* < 0.001) with empathy, teamwork, and the nursing work environment. Hierarchical regression analysis was used to identify factors influencing person-centered care among psychiatric nurses. The analysis revealed that the nursing work environment (*p* < 0.001), teamwork (*p* < 0.001), empathy (*p* < 0.001), type of hospital (general hospital) (*p* = 0.002), and age (*p* = 0.037) significantly influenced person-centered care, explaining 78.7%. Conclusions: Enhancing PCC among psychiatric nurses requires the development of training and educational programs that bolster empathy and teamwork. Additionally, improvements and strategic enhancements to the nursing work environment are essential.

## 1. Introduction

Person-centered care (PCC) emphasizes the protection of dignity and autonomy for care recipients, respects their choices, and provides individualized care, treating them as independent individuals [1,2]. In this model, nurses are expected to go beyond routine tasks such as administering medications and performing interventions based on nursing processes. They are also encouraged to adopt a holistic approach. This includes actively listening to and empathizing with the individual needs of patients, involving them in their own care, and fostering communication through a therapeutic alliance [3]. Implementing PCC not only enhances patient satisfaction, health outcomes, and quality of life, but also boosts organizational commitment and job satisfaction among staff, ultimately benefiting organizational performance [1,2].

Recently, there has been a growing movement to ensure the autonomy and self-determination of individuals with mental illnesses, promoting their human rights. The World Health Organization (WHO) has published guidelines for community mental health services under the title “Promoting Person-Centered and Rights-Based Approaches” [4]. Similarly, in South Korea, the Mental Health Welfare Act was revised in 2016 [5], and the Human Rights Report on Persons with Mental Disabilities was published by the National Human Rights Commission in 2021 [6]. These documents emphasize the human rights of individuals with mental illnesses. In line with these contemporary trends, PCC is a crucial concept, as it involves care recipients in decision-making about their treatment plans and empowers them as partners in care [7].

McCormack and McCance’s theory of PCC [1] outlines four components: the prerequisites of nurses, the PCC environment delivered by nurses to patients, the patient-centered processes involving various activities, and the patient-centered outcomes achieved through effective PCC. This theory suggests that effective person-centered outcomes can be realized when both individual nurse factors and organizational and environmental elements that support PCC are considered. Therefore, to implement PCC for individuals with mental illnesses, it is essential to consider all personal, organizational, and environmental factors that influence psychiatric nurses’ ability to deliver such care.

Empathy, as a personal factor influencing PCC, involves recognizing and re-experiencing another person’s experiences from their perspective, while simultaneously understanding and analyzing those experiences [8]. Active listening and self-awareness can enhance empathy, which in turn positively impacts the ability to deliver PCC [9]. Previous studies have shown that empathy-driven PCC not only reduces anxiety in surgical patients but also improves their recovery and satisfaction [10]. Additionally, in the context of dementia care, it has been demonstrated to improve the well-being of both patients and caregivers [11]. PCC based on empathy entails listening to patients’ personal emotions and anxieties, providing adequate information, and delivering personalized care that takes individual feelings into account. This approach is expected to be effective in implementing PCC for individuals with mental illnesses.

Teamwork, as an organizational factor influencing PCC, is fundamental to delivering high-quality patient care. It involves communication, collaboration, trust, respect, shared decision-making, accountability, and flexibility among healthcare professionals [12]. Teamwork enhances capabilities, efficiency, and innovation, thereby boosting nurses’ confidence in providing PCC, reducing sensitive incidents/errors in nursing, and improving the quality of care [13]. It has been reported that higher levels of teamwork are associated with increased empathy, which, in turn, enhances the performance of PCC [14]. Active teamwork within an organization positively influences team adaptability and shifts in the perception of PCC [15]. Therefore, the relationship between teamwork and PCC in the mental health nursing field should be closely examined.

The nursing work environment, as a factor influencing PCC, is a comprehensive concept that includes not only the material environment but also the human environment, such as nurses’ participation in hospital policies, co-operative relationships with medical staff, the capabilities of the nursing manager, and adequate staffing—essential elements for providing quality nursing [16]. Organizational support for high-quality nursing is linked to positive outcomes for both patients and nurses [17]. A positive nursing work environment is crucial for providing individualized care, highlighting the significance of a therapeutic nursing environment in the PCC delivery process [18]. However, in South Korea’s psychiatric healthcare institutions, nurses encounter challenging work environments characterized by staff shortages, which hinder effective responses to emergencies such as self-harm or harm to others. Additionally, a culture that often dismisses violence, sexual harassment, and verbal abuse by patients as mere symptoms leads to psychological stress for nurses, who must continue working in the same ward as aggressive patients [19]. These elements of the nursing work environment are shaped by the healthcare delivery system, economic level, and cultural context of each country, underscoring the need to consider these factors when exploring the relationship between the nursing work environment and PCC.

Reviewing previous studies related to PCC reveals numerous investigations focused on chronic disease patients, dementia patients, and surgical patients, examining the implementation of PCC and its influencing factors [10,11,20]. However, despite increasing recognition of the importance of ensuring autonomy and self-determination for individuals with mental illnesses and promoting their human rights through PCC, research on PCC provided by psychiatric nurses remains insufficient. Therefore, this study aims to explore the relationship between empathy, teamwork, the nursing work environment, and PCC among nurses working in psychiatric hospitals, and to identify the factors influencing PCC. The findings are intended to provide foundational data for developing strategies and interventions to enhance the implementation of PCC by psychiatric nurses in hospital settings.

## 2. Materials and Methods

### 2.1. Study Design, Participants

This study is descriptive correlational research aimed at examining the impact of empathy, teamwork, and the nursing work environment on PCC among psychiatric nurses. The criteria for participants are as follows: nurses working in psychiatric wards in Korea with at least one year of experience in such settings at university hospitals, general hospitals, national and public psychiatric hospitals, or psychiatric specialty hospitals in South Korea. This criterion is based on previous research [21] indicating that at least one year of experience is necessary to gain sufficient expertise as a psychiatric nurse. Additionally, participants were selected based on their understanding of the study’s objectives and their consent to participate. The sample size was calculated using the G*Power 3.1.9 program, based on a previous study [22] which investigated the PCC for intensive care nurse, assuming an effect size of 0.15, a significance level of 0.05, a power of 0.08, and 15 independent variables, which indicated a minimum requirement of 139 participants. To account for a potential 20% dropout rate, data from 167 participants were ultimately collected.

### 2.2. Procedures

After approval by the Institutional Review Board of K University (KHSIRB-23-464), a recruitment notice for research participants was posted on an online community site for psychiatric nurses. The post included the purpose of the study, the criteria for selecting subjects, and the contact information of the researcher, who could answer any questions if necessary. Subsequently, only those subjects who agreed to participate were asked to access an additional link and complete a questionnaire. The questionnaire took approximately 20 min to complete. All questionnaires were accessed through the same link, and anonymity was maintained. All questions were set as mandatory to prevent missing items. The post was published from 11 to 19 January 2024, and final questionnaires were collected from 167 research subjects who submitted completed forms. Duplicate responses were identified through the contact information collected for participant incentives, and a total of 167 responses were used for analysis.

### 2.3. Measurements

Empathy was measured using the Korean Version of the Jefferson Empathy Scale for Health Professionals (K-JSE-Hp), which was developed by Hojat et al. [23] for healthcare professionals and validated for nurses by Ryu and Bang [24]. This tool consists of 18 items across three sub-domains: perspective-taking (10 items), compassionate care (6 items), and standing in the patient’s shoes (2 items). Each item is rated on a 7-point Likert scale, ranging from “strongly disagree” (1 point) to “strongly agree” (7 points), with higher scores indicating greater empathy. Cronbach’s α was 0.89 in the study by Ryu and Bang [24].

Teamwork was assessed using the Korean Version of the tool developed by Larson and LaFasto [25], with contextual modifications for nursing by Cho [26]. This instrument includes 23 items across three sub-domains: shared goals (5 items), results orientation (7 items), and mutual co-operation (11 items). It uses a 5-point Likert scale, with higher scores indicating greater teamwork. Cronbach’s α was 0.96 in Cho’s study [26].

The nursing work environment was measured using the Korean version of the tool developed and validated by Ko [27]. This instrument consists of 20 items across three sub-domains: safety management system (8 items), nursing manager’s competence (6 items), and nurse support system (6 items), rated on a 4-point Likert scale. Higher scores indicate a better perceived nursing work environment. Cronbach’s α was 0.90 in Ko’s study [27].

PCC was measured with the Korean version of the Person-Centered Practice Inventory-Staff (PCPI-S), validated by Kim and Tak [28] from the original developed by Slater et al. [29]. It comprises 51 items across three sub-domains: prerequisites (13 items), person-centered care environment (22 items), and person-centered care process (16 items), rated on a 5-point Likert scale. Higher scores signify a higher level of PCC. The Cronbach’s α was 0.95 in Kim and Tak’s study [28].

### 2.4. Statistical Analysis

The data collected in this study were statistically analyzed using the SPSS/WIN 26.0 program. Differences in empathy, teamwork, nursing work environment, and PCC were assessed based on participants’ general characteristics. This analysis involved calculating means and standard deviations and conducting *t*-tests, one-way ANOVA, and Pearson’s correlation coefficients. Post-hoc testing was performed using the Scheffe test. All tools’ reliability, measured as internal consistency, was evaluated using Cronbach’s alpha.

Additionally, correlations between empathy, teamwork, nursing work environment, and PCC were explored using Pearson’s correlation coefficients. Factors influencing PCC were identified through hierarchical regression analysis. In Model 1, factors that showed significant differences from PCC among general characteristics were entered. The independent variables were age, marital status, type of medical institution, total clinical experience, work type, certification status, and the number of patients per nurse. For categorical variables, marital status was dummy-coded with “single” as the reference group. The reference category was “psychiatric hospitals” for medical institutions, “fixed shifts” for work type, “no certification” for certification status, and “fewer than six patients” for the number of patients per nurse. In Model 2, empathy, an individual factor, was entered; in Model 3, teamwork, an interpersonal factor; and in Model 4, nursing work environment, an organizational factor. Tolerance and VIF were checked to evaluate multicollinearity.

## 3. Results

### 3.1. Characteristics of Participants

A total of 167 psychiatric nurses, representing a representative sample, participated in the study; 94.0% of them were women. The average age of the participants was 32.85 ± 5.24 years. Among the nurses, 76.0% were unmarried. In terms of educational background, 88.0% held a bachelor’s degree. The majority of participants worked in public psychiatric hospitals, accounting for 37.7% of the employment settings. The average length of experience in psychiatric nursing was 38.29 ± 25.45 months, and total clinical experience was 79.71 ± 47.28 months (Table 1).

### 3.2. Differences in PCC According to the General Characteristics of Participants

The analysis of differences in PCC based on the general characteristics of participants revealed significant differences according to age (F = 3.59, *p* = 0.015), marital status (t = 5.27, *p* < 0.001), type of medical institution (F = 27.29, *p* < 0.001), total clinical experience (F = 4.58, *p* = 0.012), work schedule (F = 7.83, *p* = 0.001), certification (mental health nurse or psychiatric nurse practitioner) status (t = 5.55, *p* < 0.001), and the number of patients per nurse per shift (F = 6.26, *p* < 0.001) (Table 1).

### 3.3. Reliability and Correlations Among Empathy, Teamwork, Nursing Work Environment, and PCC in Psychiatric Nurses

The Cronbach’s alpha was 0.92 in empathy, 0.96 in teamwork, 0.91 in nursing working environment, and 0.97 in PCC.

Empathy (r = 0.64, *p* < 0.001), teamwork (r = 0.79, *p* < 0.001), and nursing work environment (r = 0.79, *p* < 0.001) in psychiatric nurses all showed positive correlations with PCC (Table 2).

### 3.4. Factors Influencing PCC in Psychiatric Nurses

A hierarchical regression analysis was conducted to examine the factors influencing PCC among psychiatric nurses. In Model 1, general characteristics were included; Model 2 incorporated empathy; Model 3 focused on teamwork; and Model 4 added the nursing work environment to assess their impacts on PCC. Across all models (Models 1–4), the tolerance exceeded 0.1, and the variance inflation factor (VIF) varied between 1.28 and 4.65, all values being under 10, which indicates that there were no multicollinearity issues among the variables.

Model 1 explained 36.2% of the variance in PCC. Compared to psychiatric hospitals, the levels of PCC were significantly higher at advanced general hospitals (β = 0.28, *p* = 0.002) and public psychiatric hospitals (β = 0.41, *p* < 0.001). Additionally, nurses with certifications (β = 0.41, *p* < 0.001) and those caring for fewer than six patients per shift, as compared to six to 14 patients (β = 0.30, *p* < 0.001), also demonstrated significantly higher PCC.

Model 2, which included empathy, showed an 18% increase in explanatory power compared to Model 1, with a total of 53.9%. Model 3, which further included teamwork, saw a 20% increase in explanatory power compared to Model 2, bringing the total to 73.9%.

Model 4, which added the nursing work environment, demonstrated a 5% increase in explanatory power compared to Model 3, reaching a total of 78.7%. Among the general characteristics, age (β = 0.14, *p* = 0.037) and advanced general hospitals (β = 0.17, *p* = 0.002) were found to significantly influence PCC. Furthermore, empathy (β = 0.27, *p* < 0.001) and teamwork (β = 0.35, *p* < 0.001), which were previously found to be significant, and the nursing work environment (β = 0.39, *p* < 0.001), also had significant impacts on PCC (Table 3).

## 4. Discussion

This study aimed to analyze the relationships among empathy, teamwork, the nursing work environment, and PCC among psychiatric nurses. It also sought to identify the factors that influence PCC. The goal was to provide foundational data for developing strategies and interventions to enhance PCC performance among psychiatric nurses in hospital settings.

The findings of the study indicated that the nursing work environment, teamwork, and empathy significantly influenced PCC. The average PCC score among the psychiatric nurses in this study was 4.02, higher than that of general hospital nurses (3.50) [30] and university hospital nurses (3.65) who used the same measurement tool [31]. This higher score among psychiatric nurses may be attributed to their focus on patients’ rights and recovery, their recognition of patients as unique individuals, and their role in providing personalized responses and care, which distinguishes them from general nurses [7,19].

Based on the analysis of differences in PCC according to general characteristics, it was found that individual factors such as age, marital status, and total clinical experience influence PCC levels. Notably, PCC was higher among those aged 25–29 compared to those over 40, and higher among those with fewer than 5 years of total clinical experience compared to those with more than 10 years. These findings are consistent with studies conducted on intensive care unit (ICU) nurses [22], but they contrast with research suggesting that PCC levels increase with age [31]. Two explanations can be proposed for these observations. First, as nurses accumulate more clinical experience, job stress may also increase, potentially hindering their ability to provide PCC [32,33]. Second, more experienced and older nurses are more likely to assume managerial roles rather than engage directly in patient care, leading to younger and less experienced nurses demonstrating a greater interest in PCC. Further research is necessary to confirm these findings.

In addition to individual factors, PCC also varies according to work environment factors such as the number of patients assigned per shift, the type of healthcare institution, and work patterns. This aligns with previous studies that have shown PCC is higher when nurses manage fewer patients [22,34]. When the number of patients per shift is high, nurses face an excessive workload, which can lead to burnout and other issues such as turnover and staff shortages. However, ensuring adequate nursing staff not only mitigates burnout from heavy workloads but also facilitates personalized care for each patient. This, in turn, can shorten hospital stays and positively affect patient safety and health outcomes [35]. Therefore, to enhance PCC, it is crucial to consider these work environment factors. Additionally, PCC was significantly higher among those with mental health professional certifications or psychiatric mental health nursing practitioner certifications. This is likely because the education related to these certifications and the continuing education required to maintain them have enhanced sensitivity to the rights of individuals with mental illnesses, as well as improved communication skills and various psychotherapeutic techniques [1]. Therefore, to improve person-centered care, ongoing education and programs in various areas such as human rights, communication, and psychotherapy techniques are necessary.

To examine the impact of empathy, teamwork, and the nursing work environment on PCC among psychiatric nurses, a hierarchical regression analysis was conducted, controlling for general characteristics. The analysis revealed that the nursing work environment was the most significant factor influencing PCC among psychiatric nurses. This finding is consistent with research conducted on nurses in long-term care hospitals [36] and general hospitals [37], which also showed that the nursing work environment affects PCC. Furthermore, the study indicated that tertiary hospitals had a more substantial impact on PCC compared to psychiatric specialty hospitals. This is likely due to higher satisfaction with the nursing work environment in tertiary hospitals [38], which facilitates more effective PCC. Therefore, it is essential to enhance the nursing work environment at an organizational level to improve PCC among psychiatric nurses.

The second factor influencing PCC among psychiatric nurses was identified as teamwork. This finding is consistent with previous studies involving ICU nurses [39] and general hospital nurses [14], which reported that teamwork positively affects PCC. It also aligns with the assertion by McCormack and McCance [1] that PCC can occur within collaborative relationships among healthcare staff. In situations involving a range of psychiatric symptoms, such as aggression, manipulation, self-harm, or suicidal behavior, therapeutic responses necessitate the collaboration of various healthcare professionals, including doctors, nurses, and caregivers. High-quality teamwork ensures that patients receive superior PCC [40]. Therefore, the implementation of various systems, programs, and supervisory measures to strengthen teamwork is necessary.

The third factor influencing PCC among psychiatric nurses was empathy. This finding aligns with previous research involving ICU [39] and general hospital nurses [14], which found that empathy affects PCC. Empathy is essential for delivering PCC, particularly for psychiatric patients who may experience a range of symptoms including hallucinations, delusions, anxiety, depression, loss of control, and risks of self-harm or harm to others [11]. It allows nurses to tailor their care to the unique situations, emotions, and symptoms of each patient. Given that empathy, coupled with communication training, can enhance PCC among psychiatric nurses, future research should concentrate on developing related educational programs or initiatives.

This study has several limitations. First, the findings may not be generalizable as the sample included some psychiatric hospital nurses who were recruited by convenience sampling and completed an online survey. It is recommended that future studies increase the sample size to validate the results. Second, this study identified only a limited number of variables influencing PCC, which may restrict the scope of the findings. Future research should encompass a wider array of individual and work environment variables that could affect PCC.

## 5. Conclusions

This study identified the nursing work environment, teamwork, and empathy as influential factors affecting PCC among psychiatric nurses. Based on these findings, future efforts should concentrate on developing educational and training programs aimed at enhancing individual empathy skills in nurses and improving teamwork among healthcare providers. Additionally, it is crucial to formulate strategies that enhance the nursing work environment, taking into account the unique challenges of psychiatric nursing.

## Figures and Tables

**Table 1:** Difference in study variables according to general characteristics (*N* = 167).

Variables	Categories	n (%) /M ± SD	Person-Centered Care			
			M ± SD	t/F	*p*	Scheffe
Gender	Male	10 (6.0)	4.04 ± 0.51	0.11	0.909	
	Female	157 (94.0)	4.02 ± 0.50			
Age		32.85 ± 5.24	4.02 ± 0.50	−2.32	0.022 **	
Religion	Yes	54 (32.3)	4.04 ± 0.49	0.26	0.794	
	No	113 (67.7)	4.01 ± 0.50			
Marital status	Unmarried	127 (76.0)	4.11 ± 0.51	5.27	<0.001 ***	
	Married	40 (24.0)	3.75 ± 0.32			
Education	Associate’s degree	12 (7.2)	4.10 ± 0.37	0.52	0.595	
	Bachelor’s degree	147 (88.0)	4.02 ± 0.51			
	≥Master’s degree	8 (4.8)	3.87 ± 0.33			
Type of medical institution	General hospital ^a^	57 (34.1)	4.04 ± 0.45	27.29	<0.001 ***	c < a < b
	Public hospital ^b^	63 (37.7)	4.27 ± 0.41			
	Psychiatric hospital ^c^	47 (28.2)	3.66 ± 0.45			
Total psychiatric clinical experience (months)		38.29 ± 25.45	4.02 ± 0.50	−1.25	0.212	
Total clinical experience (months)		76.71 ± 47.28	4.02 ± 0.50	−2.47	0.014 **	
Position	General nurse ^a^	147 (88.0)	4.05 ± 0.51	1.63	0.198	
	Charge nurse ^b^	15 (9.0)	3.81 ± 0.38			
	Head nurse ^c^	5 (3.0)	3.94 ± 0.34			
Work schedule	3-shift rotation ^a^	125 (74.9)	4.10 ± 0.51	7.83	0.001 **	c < a
	2-shift rotation ^b^	10 (6.0)	4.02 ± 0.35			
	Fixed shift ^c^	32 (19.1)	3.72 ± 0.35			
Certification status	Yes	122 (73.1)	4.14 ± 0.44	5.55	<0.001 ***	
	No	45 (26.9)	3.70 ± 0.51			
Number of patients per nurse per shift	<6 ^a^	11 (6.6)	4.29 ± 0.53	6.26	<0.001 ***	d < c,a
	6–13 ^b^	102 (61.1)	3.97 ± 0.48			
	14–21 ^c^	42 (25.1)	4.19 ± 0.49			
	≥22 ^d^	12 (7.2)	3.61 ± 0.26			

*** *p* < 0.001, ** *p* < 0.05. Sub-categories are labeled a, b, c and d based on the Scheffe test.

**Table 2:** Reliability and correlation of Empathy, Teamwork, Nursing Working Environment, PCC.

Variable	M ± SD	Empathy	Teamwork	Nursing Work Environment	PCC
Empathy	5.46 ± 0.66	1			
Teamwork	3.98 ± 0.60	0.50 (<0.001)	1		
Nursing work environment	3.28 ± 0.42	0.44 (<0.001)	0.78 (<0.001)	1	
PCC	4.02 ± 0.50	0.64 (0.001)	0.79 (<0.001)	0.79 (0.001)	1

**Table 3:** Factors influencing person-centered care by psychiatric nurses (*N* = 167).

	Model 1				Model 2				Model 3				Model 4			
	B	SE	β	*p*	B	SE	β	*p*	B	SE	β	*p*	B	SE	β	*p*
Age	−0.00	0.01	−0.05	0.686	0.01	0.01	0.12	0.237	0.01	0.01	0.15	0.051	0.01	0.01	0.14	0.037
Married	−0.17	0.10	−0.15	0.089	−0.05	0.09	−0.04	0.568	−0.07	0.06	−0.06	0.261	−0.05	0.06	−0.04	0.379
Tertiary hospital	0.30	0.09	0.28	0.002	0.23	0.08	0.22	0.004	0.26	0.06	0.25	<0.001	0.17	0.06	0.17	0.002
Public psychiatric hospital	0.41	0.09	0.41	<0.001	0.16	0.09	0.16	0.067	0.16	0.07	0.15	0.017	0.10	0.06	0.09	0.109
Total clinical experience(months)	0.00	0.00	0.12	0.312	−0.00	0.00	−0.14	0.164	−0.00	0.00	−0.05	0.515	0.00	0.00	0.02	0.783
3-shift rotation	0.16	0.10	0.14	0.113	0.09	0.08	0.08	0.278	−0.01	0.06	−0.01	0.868	0.01	0.06	0.01	0.884
2-shift rotation	0.21	0.16	0.10	0.197	0.18	0.13	0.09	0.188	0.03	0.10	0.02	0.744	0.00	0.09	0.00	0.998
Certified (mental health nurse or Psychiatric nurse practitioner)	0.33	0.07	0.30	<0.001	0.24	0.06	0.22	<0.001	0.09	0.05	0.08	0.076	0.07	0.05	0.07	0.104
6–13 patients per nurse per shift	−0.31	0.13	−0.30	0.017	−0.14	0.11	−0.14	0.213	0.3	0.09	0.03	0.720	0.12	0.08	0.12	0.132
14–21 patients per nurse per shift	−0.07	0.14	−0.07	0.601	−0.05	0.12	−0.04	0.696	0.01	0.09	0.00	0.959	0.12	0.08	0.11	0.150
≥22 patients per nurse per shift	−0.20	0.19	−0.11	0.283	−0.29	0.16	−0.15	0.068	0.07	0.13	0.04	0.560	0.15	0.11	0.08	0.204
Empathy					0.39	0.05	0.52	<0.001	0.23	0.04	0.31	<0.001	0.21	0.04	0.27	<0.001
Teamwork									0.50	0.05	0.60	<0.001	0.29	0.05	0.35	<0.001
Nursing work environment													0.46	0.08	0.39	<0.001
F-Change (*p*) R^2^ (adjusted R^2^) *Adj* R^2^ change	9.57 (<0.001) 0.40 (0.362)				17.15 (<0.001) 0.57 (0.539) 0.177				37.09 (<0.001) 0.76 (0.739) 0.200				44.92 (<0.001) 0.81 (0.787) 0.048			

Reference: Unmarried, Psychiatric Specialty Hospital, Fixed Shift, Uncertified, <6 patients per nurse per shift, Durbin–Watson = 1.719, VIF = 1.28–4.65.

## Data Availability

The raw data supporting the conclusions of this article will be made available by the authors upon reasonable request after signing a confidentiality agreement.
